# The role of GPCRs in bone diseases and dysfunctions

**DOI:** 10.1038/s41413-019-0059-6

**Published:** 2019-07-08

**Authors:** Jian Luo, Peng Sun, Stefan Siwko, Mingyao Liu, Jianru Xiao

**Affiliations:** 1 East China Normal University and Shanghai Changzheng Hospital Joint Research Center for Orthopedic Oncology, Shanghai Key Laboratory of Regulatory Biology, Institute of Biomedical Sciences and School of Life Sciences, East China Normal University, 200241 Shanghai, China; 2 The Key Laboratory of Adolescent Health Assessment and Exercise Intervention of the Ministry of Education, East China Normal University, 200241 Shanghai, China; 3 Department of Molecular and Cellular Medicine, Institute of Biosciences and Technology, Texas A&M University Health Science Center, Houston, TX 77030 USA; 4 East China Normal University and Shanghai Changzheng Hospital Joint Research Center for Orthopedic Oncology, Department of Orthopedic Oncology, Shanghai Changzheng Hospital, Second Military Medical University, Shanghai, China

**Keywords:** Bone quality and biomechanics, Osteoporosis

## Abstract

The superfamily of G protein-coupled receptors (GPCRs) contains immense structural and functional diversity and mediates a myriad of biological processes upon activation by various extracellular signals. Critical roles of GPCRs have been established in bone development, remodeling, and disease. Multiple human GPCR mutations impair bone development or metabolism, resulting in osteopathologies. Here we summarize the disease phenotypes and dysfunctions caused by GPCR gene mutations in humans as well as by deletion in animals. To date, 92 receptors (5 glutamate family, 67 rhodopsin family, 5 adhesion, 4 frizzled/taste2 family, 5 secretin family, and 6 other 7TM receptors) have been associated with bone diseases and dysfunctions (36 in humans and 72 in animals). By analyzing data from these 92 GPCRs, we found that mutation or deletion of different individual GPCRs could induce similar bone diseases or dysfunctions, and the same individual GPCR mutation or deletion could induce different bone diseases or dysfunctions in different populations or animal models. Data from human diseases or dysfunctions identified 19 genes whose mutation was associated with human BMD: 9 genes each for human height and osteoporosis; 4 genes each for human osteoarthritis (OA) and fracture risk; and 2 genes each for adolescent idiopathic scoliosis (AIS), periodontitis, osteosarcoma growth, and tooth development. Reports from gene knockout animals found 40 GPCRs whose deficiency reduced bone mass, while deficiency of 22 GPCRs increased bone mass and BMD; deficiency of 8 GPCRs reduced body length, while 5 mice had reduced femur size upon GPCR deletion. Furthermore, deficiency in 6 GPCRs induced osteoporosis; 4 induced osteoarthritis; 3 delayed fracture healing; 3 reduced arthritis severity; and reduced bone strength, increased bone strength, and increased cortical thickness were each observed in 2 GPCR-deficiency models. The ever-expanding number of GPCR mutation-associated diseases warrants accelerated molecular analysis, population studies, and investigation of phenotype correlation with SNPs to elucidate GPCR function in human diseases.

## Introduction

**Fig. 1:**
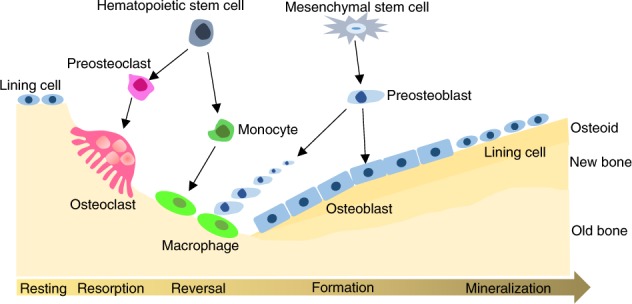
Bone cells and bone remodeling. Bone is continuously remodeled to maintain tissue integrity. Remodeling begins with old bone resorption by osteoclasts, which differentiate from hematopoietic stem cells. Following resorption, unclassified macrophage-like cells, which are also from hematopoietic stem cells, are found at the remodeling site in the intermediate or reversal phase. Osteoblast precursors, which arise from mesenchymal stem cells, are then recruited and proliferate and differentiate into mature osteoblasts and secrete new bone matrix. The matrix then mineralizes to generate new bone, completing the remodeling process

Bone development and bone remodeling are processes primarily governed by osteoblast, osteoclast, and chondrocyte differentiation and activity. Fetal bone development proceeds through two courses, intramembranous ossification (typical in flat bone formation) and endochondral ossification (primarily in long bones). Intramembranous ossification is largely influenced by mesenchymal cell differentiation into mature osteoblasts,^1^ while endochondral ossification is driven by mesenchymal cell differentiation into chondrocytes, which then undergo hypertrophy.^2^ Bone remodeling occurs throughout life and involves resorption of mature bone tissue by osteoclasts, which differentiate from hematopoietic cell precursors,^3,4^ and new bone tissue formation by osteoblasts, which arise from mesenchymal stem cells (MSCs)^5,6^ (Fig. 1). Each cell type is regulated by assorted hormones and paracrine factors. These factors determine the relative rates of bone formation and resorption, processes whose homeostasis is critical to prevent bone structure damage, and consequent metabolic bone diseases.^7^

G protein-coupled receptors (GPCRs) are the most numerous transmembrane (TM) protein family implicated in multiple biological processes, including bone development and remodeling,^8,9^ vision,^10^ taste,^11^ smell,^12^ neurotransmitter signaling,^13^ inflammation/immune response,^14^ autonomic nervous system regulation,^15^ homeostasis maintenance,^16^ and tumor growth and metastasis.^17^ Because GPCRs play important roles in physiological and pathological processes, have easily targeted ligand-binding domains, and bind diverse chemical modulators, they comprise the most important class of drug targets, accounting for 12% of all human protein drug targets and the therapeutic effects of approximately 34% of clinically used drugs.^18,19^ Certain GPCRs and their signaling pathways are responsible for bone homeostasis, and disruption or mutation of these GPCRs results in human bone diseases or dysfunctions,^20–29^ the majority of whose phenotypes have been validated in mouse models.^8,30–43^ Therefore, GPCRs are necessary for regulating bone development and remodeling.

More than 800 human GPCRs (approximately 2%–3% of all human genes) have been identified that share common structural motifs. Approximately 150 putative human GPCRs have still unknown functions with unknown ligands and are consequently called orphan receptors. A frequently used GPCR classification system designates classes by letters A–F, with subclasses designated with roman numerals.^44,45^ The A–F system was developed from known vertebrate and invertebrate GPCRs. Several groups have no human members; others contain a handful of receptors from only one single class of a species; there are even GPCRs that fail to fit into any of these six groups. Recently, a system that groups human GPCRs into five main families (glutamate (G), rhodopsin (R), adhesion (A), frizzled/taste2 (F), and secretin (S), hence the GRAFS classification system) has been proposed based on phylogenetic analysis.^46^ In this review, we use the GRAFS classification system.

## Signaling background

**Fig. 2:**
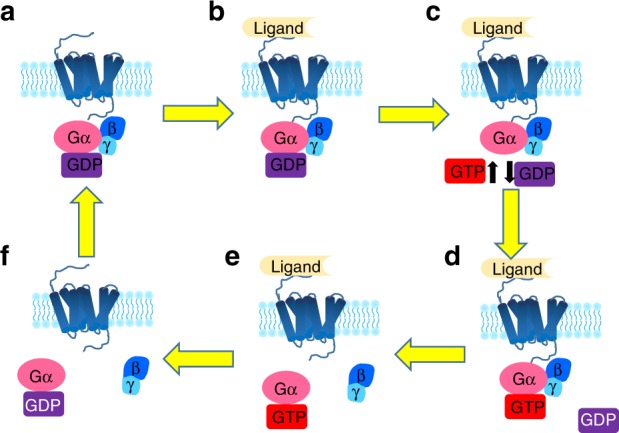
Activation cycle of G proteins/G protein-coupled receptor (GPCR) upon ligand binding. The receptor in an unbound state is inactive (**a**), and its coupled G protein is bound to GDP. Ligand binding to its GPCR (**b**) induces a change in GPCR conformation that promotes GDP exchange for GTP on the heterotrimeric complex α subunit (**c**, **d**). Both active, GTP-bound Gα and the Gβγ dimer then stimulate downstream effectors (**e**). When the ligand is no longer bound to the GPCR and the GTP on Gα is hydrolyzed to GDP (**f**), a new inactive GDP-bound heterotrimeric G protein can couple to the GPCR, and the original receptor is restored

The structural hallmark of GPCRs is the TM helical domain that transverses the cell membrane seven times. Different GPCRs can recognize diverse ligands, including ions, amines, nucleotides, peptides, proteins, lipids, organic odorants, and photons,^47^ normally using an extracellular ligand-binding domain. The cytoplasmic portion of GPCRs possesses a highly dynamic intracellular cleft where signaling partners interact with the receptor. Three families of proteins (heterotrimeric G proteins, GPCR kinases (GRKs), and arrestins)^48,49^ (Fig. 2) are the primary signaling effectors of most GPCRs.

Heterotrimeric G proteins are key transducers of GPCR signaling.^50^ Heterotrimeric G proteins have alpha (α), beta (β), and gamma (γ) subunits;^51^ β and γ remain associated throughout the signaling cycle and are referred to as the Gβγ dimer. Alpha (α) G proteins are allocated to four main classes according to the Gα sequence: Gαs, Gαi/o (Gαi1–3, GαoA,B, Gαz), Gαq (Gαq, Gα11, Gα14,16), and Gα13 (Gα12, Gα13).^52,53^ Inactive G proteins bind GDP with its Gα subunit. GPCR activation conformationally shifts the bound G protein, causing GDP exchange for GTP by the Gα subunit. The GTP-bound Gα subunit then dissociates from the Gβγ dimer (Fig. 2). Free Gα can activate effector molecules, such as adenylyl cyclase (AC). The free Gβγ dimer can also activate effectors such as potassium channels or phospholipase for downstream signaling.^54,55^

GRKs are included in the AGC kinase family (protein kinases A, G, and C).^56^ GRK family proteins share a common structure featuring a kinase domain in the loop separating α-helices 9 and 10 of the regulatory G protein signaling homology domain. Sequence homology is used to subdivide GRKs into the rhodopsin kinase subfamily (GRK1 and GRK7), the β-adrenergic receptor kinase subfamily (GRK2 and GRK3), and the GRK4 subfamily (GRK4, GRK5, and GRK6).^57^ GRK 1 and 7 expression is limited to the retina; GRK 2, 3, 5, and 6 are expressed ubiquitously; and GRK4 expression is predominantly observed in the brain, kidney, and testes.^58^ GRKs terminate GPCR activation via phosphorylation of substrate intracellular loops and C-terminal tails. The phosphorylated GPCR then binds arrestins, which exclude G protein interaction and induce receptor–arrestin complex internalization, shutting down signal transduction.^59,60^ Therefore, modulation of GRK protein stability is a potential feedback mechanism for regulating GPCR signaling and basic cellular processes.

Arrestin family proteins regulate GPCR signal transduction^61,62^ by terminating G protein signaling and initiating arrestin-mediated GPCR downstream cascades. Mammalian cells express four arrestins: arrestin-1 (also known as visual arrestin), arrestin-2 (also known as β-arrestin 1), arrestin-3 (also known as β-arrestin-2), and arrestin-4 (also known as cone arrestin). Arrestin-1 and arrestin-4 are selectively expressed in the retina, and arrestin-2 and arrestin-3 have a broad expression pattern in various cell types. Arrestin-2 and arrestin-3 are ~80% identical in sequence and have overlapping roles in GPCR regulation.^63–66^

As GPCRs have a variety of signaling modalities that can selectively stimulate (or inhibit) intracellular signaling pathways to treat different diseases by biased signaling, which can minimize the risk of side effects,^67,68^ GPCRs have been major targets of modern therapeutics. For example, the rhodopsin family GPCR Angiotensin II (AngII) type I receptor (AT1R) has been targeted for the treatment of cardiovascular diseases.^69,70^ Recently, AT1R was shown to activate both Gαq signaling and β-arrestin signaling to exert different functions and side effects. Therefore, the β-arrestin-biased ligand TRV027 for AT1R is currently in a phase II clinical trial. TRV027 specifically activates AT1R-β-arrestin signaling (associated with increased cardiomyocyte contractility and cardiac apoptosis prevention) but without stimulating Gαq signaling, which is linked to vasoconstriction and sodium and fluid retention.^71,72^

**Fig. 3:**
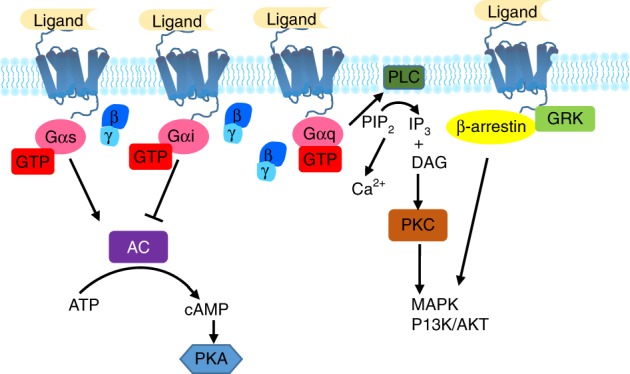
Major G protein-coupled receptor (GPCR) signaling pathways. GPCR signaling is transduced through several canonical or noncanonical pathways that ultimately proceed through second messengers. The Gs and Gi pathways converge on AC to modulate intracellular cAMP; the Gq pathway increases intracellular Ca^2+^ and MAPK and PI3K/Akt signals by activating PLC; the β-arrestin/GRK pathway activates downstream MAPK and PI3K/Akt signals. AC adenylyl cyclase, ATP adenosine triphosphate, cAMP cyclic adenosine monophosphate, PKA protein kinase A, PLC phospholipase C, PIP_2_ phosphatidylinositol 4,5-bisphosphate, IP_3_ inositol trisphosphate, DAG diacylglycerol, PKC protein kinase C, MAPK mitogen-activated protein kinase, PI_3_K phosphoinositide-3-kinase, Akt serine-threonine protein kinase, GRK G protein-coupled receptor kinase

Multiple GPCRs exhibit bone expression,^73^ and GPCR signaling regulates the proliferation, differentiation, and apoptosis of osteoblasts, osteoclasts, and chondrocytes.^6,73–76^ GPCRs signal through several canonical pathways to regulate osteoblast function^77^: the Gs and Gi pathways regulate AC, increasing or decreasing intracellular cAMP levels, respectively, while Gαq activates phospholipase C (PLC) to increase intracellular calcium.^73,78–82^ In addition, GRK phosphorylation and β-arrestin signaling govern osteoblast function^83–85^ (Fig. 3). Recent advances have shed light on the mechanisms of osteoclast^9,76,86,87^ and chondrocyte^88–92^ differentiation and function; however, how GPCR signaling regulates osteoclasts and chondrocytes remains largely unknown. The expression of multiple GPCRs by different bone cells and the activation of multiple signaling pathways by a single GPCR, together with the wide variety of GPCRs and the signaling redundancy often seen downstream of GPCR activation, pose significant challenges to clarifying a given GPCR’s function in bone development and disease. Nevertheless, incremental advances into the in vivo roles of GPCR signaling pathways and their effects on bone biology have been recently attained (Fig. 2).

## Diseases or dysfunction caused by GPCR mutation or deletion in humans and mice

### Glutamate family

Glutamate receptors are predominantly expressed by neuronal and glial cells^93^ and transmit glutamate-mediated postsynaptic excitation of neural cells. They regulate neural communication, memory formation, and learning. Several diseases in humans have an established association with glutamate receptor gene mutations, including Parkinson’s disease,^94^ Huntington’s disease,^95^ ischemic stroke seizures,^96^ attention deficit hyperactivity disorder,^97^ addiction,^98^ and autism.^99^

There are two types of glutamate receptors: metabotropic receptors (mGluRs) bearing a single 7TMD and multimeric ligand-gated ion channels, and ionotropic receptors (iGluRs).^100^ The mGluRs are linked to G protein complexes whose associated GTPase activity mediates their signaling. Upon binding glutamate, mGluRs initiate G protein activation as described above, triggering intracellular signaling cascades.^101^ The iGluRs are a composite family, including the kainate (Ka), *N*-methyl-d-aspartate (NMDA), and α-amino-3-hydroxy-5-methyl-4-isoxazole propionic acid (AMPA) groups.^102^ The different iGluRs have different properties and kinetics, with AMPA and kainates predominantly active in Na^+^ and K^+^ permeability, while NMDA is predominantly active Ca^2+^ in permeability.^100^

A variety of glutamate receptors have abundant bone expression and function in bone remodeling.^103–107^ One such receptor is an essential regulator of calcium homeostasis, the calcium-sensing receptor (CASR). Under physiological Ca^+^^2^ levels, CASR is activated by extracellular calcium and inhibits parathyroid hormone (PTH) and PTH-related protein (PTHrP) secretion. If systemic calcium levels drop, CASR signaling decreases, allowing PTH and PTHrP secretion, which induces renal retention of Ca^+2^, increased gut Ca^+2^ absorption, and eventually elevated bone resorption.^108,109^ Lorentzon et al. found that different CASR alleles are related to bone mineral density (BMD),^110^ and healthy adolescent girls with the S allele have lower BMD than individuals lacking the S allele, and Di et al.^20^ also verified that the CASR A986S polymorphism increased the risk of osteoporosis in aging males. Knockout of *Casr* in osteoblasts, driven by *2.3Col(I)-Cre* or *OSX-Cre*, resulted in reducing BMD and bone length to block mouse skeletal development.^88^ Moreover, knockout of *Casr*, driven by *Col(II)-Cre*, in chondrocytes blocks embryonic development and cartilage maturation.^88^ Additionally, the mice with global knockout of *Casr* showed a significantly reduced body length.^30^

Additional phenotypes were validated in mouse models, in which deletion of *Gababr1*,^111^ *Gprc6a*,^112,113^ and *Grm1*^114^ reduced mouse BMD, while *Tas1r3* deficiency impaired osteoclast function, resulting in reduced bone resorption and increased bone mass.^115,116^ *Gababr1*-null mice reduce BMD primarily through negatively regulating BMP and upregulating RANKL to affect bone remolding,^111^ while the effects of *Gprc6a* deletion were primarily caused by defective osteoblast-mediated bone mineralization.^112,113^ *Grm1* knockout mice exhibit enhanced bone maturation, marked by premature growth plate fusion, shortened long bones, and lower BMD^114^ (Table 1).

**Table 1:** Bone diseases or dysfunctions caused by glutamate GPCR mutation or deletion

GPCR	Species	Bone diseases or dysfunctions caused by GPCR mutation or deletion	References
CASR	Human	Association between A986S polymorphism, reduced BMD, and elevated osteoporosis risk	Lorentzon et al.^110^ Di et al.^20^
	Mouse	Reduced body length and bone mass	Ho et al.^30^ Chang et al.^88^
GABABR1	Mouse	Reduced BMD	Takahata et al.^111^
GPRC6A	Mouse	Reduced BMD, mineralization, and femur width	Pi et al.^112^ Pi et al.^113^
GRM1	Mouse	Reduced body length and BMD	Musante et al.^114^
TAS1R3	Mouse	Reduced bone resorption and increased bone mass	Eaton et al.^115^ Simon et al.^116^

*BMD* bone mineral density, *GPCR* G protein-coupled receptor

### Rhodopsin family

The rhodopsin family (class A in the A–F classification system), which includes 701 members in humans, is the largest vertebrate GPCR family and regulates many processes throughout the body. Rhodopsin receptors are structurally different from other GPCR subfamilies as they generally possess short N-termini.^47^ The ligands for most rhodopsin receptors, though diverse in structure, typically bind a cavity between the TM regions,^117^ whereas in other GPCR families, the N-terminus plays a key role in ligand binding. Important exceptions exist, particularly the glycoprotein-binding receptors (lutropin, follitropin, and thyrotropin), which bind ligands through an N-terminal domain.

Based on experimental phylogenetic investigation, there are four main groups of rhodopsin GPCRs (α, β, γ, and δ), which are subdivided into 13 subgroups in humans.^46^ The α-group includes five branches: the prostaglandin, amine, opsin, melatonin, and MECA receptor clusters. The β-group includes 36 receptors without any main branches. The γ-group contains three main branches: the SOG, MCH, and chemokine receptor clusters, while the four branches of the δ-group are the MAS-related, glycoprotein, purin, and olfactory receptor clusters.^46^

#### The rhodopsin family α-group

When the α-group rhodopsin GPCRs were analyzed for effects of mutation or deletion, eight GPCRs were associated with human bone diseases or dysfunctions. Mutations of *ADRB2*,^118^ *CNR2*,^21,119,120^ and *DRD4*^121,122^ were associated with reduced human BMD, while *MC4R*^123^ increased BMD. *ADRB2* genotypes AG and GG had more frequent osteoporosis at the femoral neck (3.27 and 3.89 times more frequent, respectively, compared to AA genotype) in a study of 592 postmenopausal Korean women.^118^ Woo et al. suggested that the *CNR2* gene polymorphisms rs2501431, rs3003336, rs2229579, and rs4237 may affect BMD in postmenopausal Korean women.^119^ A *CNR2* polymorphism is associated with low BMD in Japanese^120^ and French women.^21^ Japanese men with the 521C>T polymorphism of *DRD4* more frequently had reduced BMD, but no difference was reported in women.^121^ Five missense mutations (N62S, R165Q, V253I, C271Y, and T112M) in *MC4R* are associated with a marked increase in human BMD and a tendency toward tall height^121^ (Table 2).

**Table 2:** Bone diseases or dysfunctions caused by the α-group of rhodopsin GPCR mutation or deletion

GPCR	Species	Bone diseases or dysfunctions caused by GPCR mutation or deletion	References
A1R	Mouse	Elevated BMD and bone mass	He et al.^129^
			Kara et al.^130^
			Kara et al.^131^
A2AR	Mouse	Reduced bone mass and inhibited bone formation	Mediero et al.^139^
			Mediero et al.^140^
A2BR	Mouse	Reduced BMD and bone mass	Corciulo et al.^141^
			Carroll et al.^142^
A3AR	Mouse	Promoted osteosarcoma growth	Iyer et al.^25^
ADRB1	Mouse	Reduced bone mass and BMD	Pierroz et al.^143^
			Bonnet et al.^144^
ADRB2	Human	SNP associated with reduced BMD, increased risk of fractures, and heterotopic ossification	Lee et al.^118^
			Mitchell et al.^128^
	Mouse	Reduced bone mass and BMD	Pierroz et al.^143^
			Bonnet et al.^144^
CNR1	Mouse	Increased trabecular bone mass	Tam et al.^132^
			Idris et al et al.^133^
			Khalid et al.^134^
CNR2	Human	The rs2501431, rs3003336, rs2229579, and rs4237 polymorphisms associated with osteoporosis and decreased BMD	Woo et al.^119^
			Yamada et al.^120^
			Karsak et al.^21^
	Mouse	Reduced bone mass in C57BL/6 background	Ofek et al.^31^
			Sophocleous et al.^159^
		Increased bone mass in CD1 background	Sophocleous et al.^160^
		Reduced age-related or ovariectomy-induced bone loss	Sophocleous et al.^157^
			Idris et al.^158^
		Increased femoral and vertebral body length	Wasserman et al.^151^
DRD2	Human	The A1 allele was associated with reduced body height	Miyake et al.^22^
DRD4	Human	The 521C>T polymorphism was associated with reduced BMD	Yamada et al.^121^
EDG2	Human	The polymorphisms associated with osteoarthritis	Mototani et al.^26^
EP1	Mouse	Increased bone mass and strength	Zhang et al.^135^
		Accelerated fracture healing	Zhang et al.^153^
EP2	Mouse	Reduced bone stiffness	Akhter et al.^154^
EP4	Mouse	Inhibited bone resorption and osteoclast formation	Miyaura et al.^155^
			Sakuma et al.^156^
H4R	Human	Higher expression of H4R mRNA in osteoarthritic patient synovial tissues	Yamaura et al.^23^
	Mouse	Promoted bone destructive process of osteoporosis	Kim et al.^152^
HTR2	Mouse	Reduced bone mass and bone formation	Kumar et al.^145^
			Yadav et al.^146^
			Collet et al.^147^
LPAR1	Mouse	Reduced body length and bone mass	Gennero et al.^32^
			David et al.^148^
M3R	Mouse	Induced osteoporosis and reduced BMD	Shi et al.^122^
			Lips et al.^33^
			Kauschke et al.^34^
M5R	Mouse	Induced osteoporosis	Kauschke et al.^34^
MC1R	Mouse	Increased BMD and bone mass and accelerated osteoarthritis	Lorenz et al.^136^
MC4R	Human	Mutations N62S, R165Q, V253I, C271Y, and T112M were associated with increased BMD, and the C allele reduced fracture risk	Farooqi et al.^123^
			Gary et al.^124^
	Mouse	Increased BMD, bone mass, and strength	Ahn et al.^137^
			Braun et al.^138^
MTNR1B	Human	CT genotype was associated with AIS and osteoporosis	Moroca et al.^24^
			Li et al.^127^

*AIS* adolescent idiopathic scoliosis, *BMD* bone mineral density, *GPCR* G protein-coupled receptor, *SNP* single-nucleotide polymorphism

*DRD2* polymorphism could influence human height in childhood, acting through the hypothalamus (growth hormone (GH)-releasing hormone)–pituitary (GH)–Insulin-like growth factor 1 (IFG-1) axis,^22^ while MTNR1B polymorphism was associated with adolescent idiopathic scoliosis (AIS). Moroca et al. found that, compared with CC (*MTNR1B*) (rs4753426), the risk of AIS significantly increased in Hungarians bearing the CT allele.^24^ Gary et al. reported lower fracture incidence among elderly Swedish women bearing the *MC4R* C-allele.^124^ Curiously, lipocalin 2, a recently identified ligand of MC4R, is secreted by osteoblasts in mice and signals to suppress appetite by binding MC4R-expressing hypothalamic neurons^125^; MC4R polymorphisms have also been associated with early-onset obesity.^126^ Mutation of *CNR2*^21^ and *MTNR1B*^127^ had an additional association with human osteoporosis. Karsak et al. found that two missense variants (the double single-nucleotide polymorphism (SNP) rs2502992–rs2501432 and Gln63Arg; rs2229579 and His316Tyr) are associated with osteoporosis in postmenopausal Caucasian women,^21^ while Li et al. found that *MTNR1B* rs3781638 is associated with osteoporosis in Chinese geriatrics.^127^ The *ADRB2* polymorphism (rs1042714) was also associated with heterotopic ossification in adult trauma patients with fractures.^128^ *EDG2*^26^ and *H4R*^23^ were associated with human osteoarthritis (OA) in Japanese people. *EDG2* SNPs (rs3739708) affect AP-1 transcriptional activity, which may increase *EDG2* expression when the allele is upregulated in knee OA patients, while Yamaura et al. found higher expression of *H4R* mRNA in synovial tissues from patients with OA (Table 2).

Eighteen α-group GPCR genes have been reported to cause bone dysfunctions when deleted in mouse models. The deletion of *A1r*,^129–131^ *Cnr1*,^132–134^ *EP1*,^135^ *Mc1r*,^136^ and *Mc4r*^137,138^ increased bone mass and BMD, while *A2ar*,^139,140^ *A2br*,^141,142^ *Adrb1*,^118,143,144^ *Adrb2*,^143,144^ *Htr2*,^145–147^ *Lpar1*,^32,148^ and *M3r*^122^ reduced bone mass and BMD. *A1r*,^129–131^ *Cnr11*,^133^ and *Mc4r*^137^ knockout mouse bone mass and BMD were significantly increased, accompanied by impaired bone resorption; *Mc4r*-deficient mice also had higher CART expression, and deleting one CART allele ameliorated the bone resorption phenotype, suggesting that Mc4r function in hypothalamic neurons may regulate osteoclast function,^149^ although direct synovial and bone functions for proopiomelanocortin-derived peptides have been reported.^150^ Deletion of EP1^135^ increased bone mass and BMD by promoting osteoblast-mediated bone formation. A*2ar*,^139,140^ *A2br*,^141,142^ *Adrb1*,^118,143,144^ *Adrb2*,^143,144^ *Lpar1*,^32,148^ and *Ep1*^135^ knockout in mice induced bone loss by promoting bone resorption and suppressing bone reformation, while *Htr2* deletion suppressed osteoblast recruitment and proliferation and led to osteopenia.^147^ *Htr2*^147^ and *Ep1*^135^ also participate in regulating nervous system-mediated bone loss.

The deletion of *Cnr2* increased mouse body length by regulating growth plate chondrocyte function,^151^ while *Lpar1* reduced body length by regulating osteoblast function.^32^ Furthermore, M3R deletion caused mouse osteoporosis by altering osteoblast and osteoclast function or neuronal regulation,^33,34,122^ *H4r* deletion accelerated mouse rheumatoid arthritis by promoting osteoclastogenesis,^152^ and *Mc1r* deficiency caused an articular cartilage phenotype accompanied by accelerated surgically induced murine OA.^136^ Deletion of *A3ar* promoted mouse osteosarcoma cell proliferation, tumor formation, and metastasis, mainly by activating the protein kinase A (PKA)–Akt–nuclear factor (NF)-κB axis.^25^ *Ep1* deletion accelerated fracture repair by enhancing osteoblast differentiation,^153^ and *Ep2* deletion reduced mouse bone stiffness, which may be caused by stimulating cAMP formation, an early cellular signal that stimulates bone formation.^154^ *Ep4* deletion inhibited mouse bone resorption, though the reason is disputed, with one paper claiming it was a cAMP-dependent mechanism^155^ or through proinflammatory cytokines and lipopolysaccharides.^155,156^ *Cnr2* deletion reduced mouse age-related or ovariectomy-induced bone loss by osteoclast inhibition.^157,158^ Moreover, while *Cnr2* knockout reduced bone mass in C57BL/6 mice by regulating osteoblastogenesis and osteoclastogenesis,^31,159^ the opposite phenotype was found in CD1 mice, which had increased bone mass.^160^ These results suggest that different GPCRs have different physiological functions to regulate bone remodeling, and even the same gene may have different physiological functions regulating bone remodeling in different strains of mice (Table 2).

#### The β-group of the rhodopsin family

Analysis of the effects of rhodopsin β-group GPCR mutation or deletion uncovered 10 GPCRs associated with bone diseases or dysfunctions. Of particular interest is the ghrelin receptor, GHSR, whose mutation was associated with reduced human height.^27^ Normally, ghrelin secreted by the stomach induces appetite and regulates lipid metabolism. In 2 families with familial short stature, Pantel and coworkers identified a *GHSR* missense mutation that downregulated receptor protein levels and selectively impaired GHSR constitutive activity without affecting its response to ghrelin. In *Ghsr*-deficient mice, a reduction in BMD was caused by impaired bone formation, although the mechanism is disputed. In one report, the phenotype was due to acylated ghrelin signaling and was partially suppressed by unacylated ghrelin^161^; more recently, Gshr re-expression in the osteoblasts, but not in the osteoclasts, of Gshr^−/−^ mice was able to restore bone formation by promoting osteoblast differentiation.^162^ Additional β-group rhodopsin GPCRs implicated in human bone disorders, including *GNRHR*s,^28^ were associated with reduced human BMD and short stature, and *EDNRA* was associated with abnormal human tooth development.^163^ Homozygous partial loss-of-function mutations in *GNRHR*s caused the reduction in height and BMD through delayed puberty or isolated hypogonadotropic hypogonadism.^28^ The *EDNRA* (rs1429138) gene polymorphism affected gene expression during early craniofacial development and was associated with abnormal human tooth development.^163^

Additional phenotypes were identified in GPCR knockout mouse models. The deficiency of *Avpr1a*,^164^ *Npy1r*,^165,166^ and *Npy2r*^167–173^ increased mouse bone mass and BMD, while *Cckbr*,^174,175^ *Ghsr*,^161^ and *Npy6r*^176^ deficiency reduced bone mass and BMD. Tama et al. reported a dramatic bone mass increase in *Avpr1α*^−/−^ mice resulting from elevated bone formation and reduced resorption,^164^ while *Npy1r*^165,166^ and *Npy2r*^167–173^ mice directly regulate osteoblast activity and bone formation; BMD changes occur when these genes are deleted.^165^ In contrast, mice deficient in *Cckbr* had reduced bone mass and BMD by disrupted calcium homeostasis.^174,175^ *Npy6r* deletion in mice suppressed osteoblast numbers, osteoid surface area, and bone mineralization while stimulating osteoclast formation and bone resorption, presumably via a suprachiasmatic nucleus relay due to the narrow range of cells that expresses this receptor.^176^ Furthermore, *Oxtr* deletion caused mouse osteoporosis by inhibiting the differentiation of osteoblasts and stimulating osteoclast formation,^35^ and *Ednra* deletion caused mouse mandibular and craniofacial defects, possibly by regulating *Dlx5* and *Dlx66*, which are downstream mediators of *Ednra* signaling.^177–181^ Fracture repair was delayed while bone callus volume and callus strength decreased in osteoblast-specific *Npy1r* knockout mice,^182^ and *Gpr120* deletion promoted osteoblastic bone formation and negatively regulated osteoclast differentiation, survival, and function^183,184^ (Table 3).

**Table 3:** Bone diseases or dysfunctions caused by the β-group of rhodopsin GPCR mutation or deletion

GPCR	Species	Bone diseases or dysfunctions caused by GPCR mutation or deletion	References
AVPR1A	Mouse	Increased bone mass and BMD	Tama et al.^164^
CCKBR	Mouse	Induced osteopenia	Haffner et al.^174^
			Schinke et al.^175^
EDNRA	Human	The s1429138 SNP was associated with abnormal tooth development	Shaffer et al.^163^
	Mouse	Induced mandibular and craniofacial defects	Ruest et al.^177^
			Ruest et al.^178^
			Sato et al.^179^
			Tavares et al.^180^
			Clouthier et al.^181^
GHSR	Human	*GHSR* mutation was associated with reduced height	Pantel et al.^27^
	Mouse	Reduced bone mass	Delhanty et al.^161^
GNRHRs	Human	The mutations in *GNRHR*s were associated with reduced height and BMD	Lin et al et al.^28^
GPR120	Mouse	Decreased bone formation and increased bone resorption	Ahn et al.^183^
			Kim et al.^184^
NPY1R	Mouse	Increased bone mass and delay fracture repairing	Lee et al.^165^
			Baldock et al.^166^
			Sousa et al.^182^
NPY2R	Mouse	Increased BMD and bone mass	Baldock et al.^167^
			Baldock et al.^168^
			Shi et al.^169^
			Allison et al.^170^
			Sainsbury et al.^171^
			Sainsbury et al.^172^
			Lundberg et al.^173^
NPY6R	Mouse	Reduced bone mass	Khor et al.^176^
OXTR	Mouse	Induced osteoporosis	Tamma et al.^35^

*BMD* bone mineral density, *GPCR* G protein-coupled receptor, *SNP* single-nucleotide polymorphism

#### The rhodopsin family γ-group

Among the γ-group rhodopsin GPCRs, two GPCR gene polymorphisms were associated with human bone diseases or dysfunctions (Table 4). Eraltan et al. found *CCR2* V64I gene polymorphisms in postmenopausal women and demonstrated a positive association of *CCR2* Val/Ile and *CCR2* Val+ genotypes with osteoporosis risk.^185^ This polymorphism appears to increase CCR2 protein half-life^186^ and may also be associated with cancer risk and other diseases.^186–188^ Furthermore, Lu and coworkers discovered that three *OPRM1* SNPs (rs9479769, rs4870268, and rs1998221) were nominally associated with hip, spine, and whole-body BMD phenotypes in female American Caucasians, potentially via effects on alcohol consumption and/or estrogen signaling.^29^

**Table 4:** Bone diseases or dysfunctions caused by the γ-group of rhodopsin GPCR mutation or deletion

GPCR	Species	Bone diseases or dysfunctions caused by GPCR mutation or deletion	References
BDKRB1	Mouse	Reduced bone loss	Gonçalves et al.^190^
CCR1	Mouse	Reduced bone mass	Hoshino et al.^191^
			Taddei et al.^192^
CCR2	Human	*CCR2* Val/Ile and *CCR2* Val+genotype were associated with osteoporosis	Eraltan et al.^185^
	Mouse	Delayed fracture healing	Xing et al.^199^
		Larger and stronger tibial bones	Mader et al.^203^
CCR5	Mouse	Reduced cartilage degeneration postsurgery	Takebe et al.^204^
		Promoted alveolar bone resorption	Andrade et al.^205^
CCR6	Mouse	Reduced bone mass	Doucet et al.^193^
CCR7	Mouse	Reduced functional deficits and subchondral bone changes in the DMM model	Sambamurthy et al.^206^
CMKLR1	Mouse	Reduced bone mass and BMD in male	Zhao et al.^194^
CX3CR1	Mouse	Increased bone mass	Hoshino et al.^189^
CXCR2	Mouse	Reduced body length, bone mass, and BMD	Bischoff et al.^36^
		Reduced arthritis severity	Jacobs et al.^201^
CXCR4	Mouse	Reduced femoral length and bone mass	Zhu et al.^195^
		Reduced bone fracture healing	Kawakami et al.^200^
GPR1	Mouse	Reduced BMD and bone mass	Liet al et al.^196^
GPR142	Mouse	Reduced CAIA-induced arthritis severity	Murakoshi et al.^202^
GPR54	Mouse	Reduced bone mass	Brommage et al.^197^
MCHR1	Mouse	Induced osteoporosis	Bohlooly et al.^198^
OPRM1	Human	rs9479769, rs4870268, and rs1998221 SNPs were associated with reduced BMD and bone mass	Lu et al.^29^

*BMD* bone mineral density, *CAIA* collagen antibody-induced arthritis, DMM destabilization of the medial meniscus, *GPCR* G protein-coupled receptor, *SNP* single-nucleotide polymorphism

Fourteen genes from the γ-group GPCRs have been reported to cause bone dysfunctions in knockout mouse models. The deficiency of *Cx3cr1*^189^ increased mouse bone mass and BMD by regulating both osteoblasts and osteoclasts, while deficiency of *Bdkrb1*,^190^ *Ccr1*,^191,192^ *Ccr6*,^193^ *Cmklr1*,^194^ *Cxcr2*,^36^ *Cxcr4*,^195^ *Gpr1*,^196^ and *Gpr54*^197^ reduced bone mass and BMD. Deletion of *Bdkrb1* increased mouse bone loss and the number of osteoclasts by increasing differentiation into functional osteoclasts,^190^ and deficiency of *Ccr1*^191,192^ and *Gpr1*^196^ caused osteopenia due to decreased osteoclast and osteoblast activity. Doucet et al.^193^ found that *Ccr6*^−/−^ mice exhibited significantly decreased trabecular bone mass and reduced osteoblast numbers. Mechanistic studies indicated that Ccr6 loss delayed osteoblast marker gene expression, inhibited osteoblast differentiation, and reduced mineralization. Zhao et al.^194^ found that *Cmklr1* deficiency disrupted the balance between osteoblastogenesis and osteoclastogenesis, causing MSCs to shift from osteogenic to adipogenic differentiation and enhancing osteoclast formation and consequently lower bone mass in male mice. Zhu et al.^195^ found that osteoprecursor-specific inactivation of *Cxcr4* impaired osteoblast development and reduced postnatal bone formation, leading to a reduction in BMD and femoral length. Conversely, a decrease in BMD and body length in *Cxcr2*^*−/−*^ mice occurred despite no alteration in bone formation or bone resorption.^36^ Furthermore, the *Mchr1*^*−/−*^ mice have osteoporosis caused by elevated bone resorption resulting in a reduction in the cortical bone mass, while trabecular bone was unaffected.^198^ *Ccr2* deficiency reduced macrophage infiltration and impaired osteoclast function, thus delaying bone fracture healing,^199^ while *Cxcr4* knockout mice delayed bone fracture healing by inhibiting osteoblastogenesis.^200^ *Cxcr2* knockout mice had attenuated autoantibody-mediated arthritis caused by a function of Cxcr2 neutrophil recruitment,^201^ while *Gpr142* knockout mice showed reduced arthritis scores and disease incidence in an anti-type II collagen antibody-induced arthritis model alongside decreased inflammatory cytokine production.^202^ Mader et al. found that while *Ccr2*^*−/−*^ mice had larger and stronger bones than wild-type mice, they reported that *Ccr2* loss did not significantly protect against bone loss due to disuse or estrogen loss.^203^ *Ccr5* deletion was linked to reduced cartilage degeneration postsurgery without significant changes in the degree of synovitis and bone metabolic parameters^204^ and promoted osteoclast function in orthodontic tooth movement.^205^ Furthermore, *Ccr7* deletion reduced functional deficits and subchondral bone changes in a surgical destabilization of the medial meniscus model, suggesting that certain chemokine receptors may directly affect nociception^206^ (Table 4).

#### The δ-group of the rhodopsin family

Five human bone diseases or dysfunctions were associated with eight δ-group rhodopsin GPCR gene polymorphisms. Mutation of *LHCGR*^207–209^ was associated with reduced human height; *FSHR*,^210^ *RXFP2*,^211^ and *TSHR*^212^ mutations were associated with human osteoporosis; *OR2H1* was associated with human OA^213^; *FSHR*,^210^ *LGR4*,^214^ *RXFP2*,^215^ and *TSHR*^216^ were associated with reduced human BMD, and *FPR* mutation was associated with juvenile periodontitis (Table 5). Shenker et al.^209^ found eight different families with the same A>G base change that substitutes glycine for aspartate at LHCGR amino acid 578. This mutation elevated cAMP levels when transfected into COS-7 cells, suggesting constitutive luteinizing hormone receptor activation, and was correlated with precocious puberty and increased male height. Rendina et al.^210^ found that women with AA rs6166 (*FSHR*) had a higher postmenopausal osteoporosis risk than those carrying the GG rs6166 variant, and Ferlin et al.^210^ found that young men with a T222P mutation in *RXFP2* were at high risk of osteoporosis, while Liu et al.^212^ suggested that an SNP (C-to-G substitution at codon 727) in *TSHR* may be an osteoporosis risk factor. Two SNPs in *OR2H1* (rs1233490 and rs2746149) were suggestively associated with rheumatoid arthritis phenotypes.^213^ Furthermore, the SNP rs6166 of *FSHR* significantly influenced postmenopausal female BMD,^210^ the T222P mutation of *RXFP2* was associated with a high risk of reduced young adult BMD,^215^ and the *TSHR*-Asp727Glu polymorphism was associated with femoral neck BMD in elderly Caucasians.^216^ Finally, two *FPR* mutations were found in juvenile periodontitis patients: one thymine-to-cytosine substitution at base 329 and the other a cytosine-to-guanine substitution at base 378.^217^

**Table 5:** Bone diseases or dysfunctions caused by the δ-group of rhodopsin GPCR mutation or deletion

GPCR	Species	Bone diseases or dysfunctions caused by GPCR mutation or deletion	References
EBI2	Mouse	Increased bone mass	Nevius et al.^86^
FPRs	Human	Two mutations at bases 329 and 378 were associated with juvenile periodontitis	Gwinn et al.^217^
FSHR	Human	AA rs6166 (*FSHR*) was associated with increased osteoporosis risk in postmenopausal women	Rendina et al.^210^
GPR55	Mouse	Increased bone mass in males	Whyte et al.^220^
GPR65	Mouse	Accelerated bone loss induced by ovariectomy	Hikiji et al.^223^
GPR68	Mouse	Increased BMD	Krieger et al.^221^
GPR103	Mouse	Induced kyphosis and reduced BMD and bone mass	Baribault et al.^224^
LGR4	Human	Mutation of c.376C>T was associated with reduced BMD	Styrkarsdottir^214^
	Mouse	Reduced body length and bone mass	Luo et al.^8^
			Luo et al.^9^
LHCGR	Human	A single A>G base change at position 578 was associated with reduced male height	Soriano et al.^207^
			Bertelloni et al.^208^
			Shenker et al.^209^
OR2H1	Human	SNPs rs1233490 and rs2746149 were associated with rheumatoid arthritis	Orozco et al.^213^
P2Y1	Mouse	Reduced bone mass	Orriss et al.^225^
P2Y2	Mouse	Increased bone mass in C57BL/6 mice	Orriss et al.^225^
			Orriss et al.^232^
		Reduced bone mass in SV129 mice	Xing et al.^233^
P2Y6	Mouse	Increased BMD and bone mass	Orriss et al.^222^
P2Y7	Mouse	Reduced bone mass in mixed genetic mice (129/OlaXC57BL/6XDBA/2)	Ke et al.^234^
		Increased cortical thickness in C57BL/6 mice	Gartland et al.^235^
P2Y12	Mouse	Reduced bone loss induced by age and arthritis ovariectomy	Su et al.^226^
P2Y13	Mouse	Increase bone mass in young mice but reduced bone mass in mature mice	Wang et al.^229^
			Wang et al.^231^
PAR2	Mouse	Alleviated arthritis and prevented bone loss in periodontal disease mice	Ferrell et al.^230^
			Francis et al.^227^
PTAFR	Mouse	Lower bone loss and unchanged bone turnover in OVX mice	Hikiji et al.^42^
RXFP2	Human	T222P mutation was associated with osteoporosis and reduce BMD	Ferlin et al.^211^
			Ferlin et al.^215^
	Mouse	Reduced bone mass	Ferlin et al.^211^
			Ferlin et al.^215^
TSHR	Human	A C-to-G substitution at codon 727 was associated with osteoporosis and reduced BMD	Liu et al.^212^
			Van et al.^216^
	Mouse	Induced osteoporosis and reduced femur length and BMD	Abe et al.^37^

*BMD* bone mineral density, *GPCR* G protein-coupled receptor, *SNP* single-nucleotide polymorphism

Increasing evidence supports the FSHR subfamily member LGR4 in bone development. In humans, a rare nonsense mutation within *LGR4* (c.376C>T) is strongly correlated with diminished BMD,^214^ in accord with similar phenotypes in Lgr4^*−/−*^ mice.^8,9^ Furthermore, Lgr4 negatively regulates osteoclast differentiation by binding RANKL and downregulating RANK expression in mouse and human cells.^9^ In vitro studies support Lgr4 regulation of osteoblasts and bone MSCs.^8,218^ Mice treated with the Lgr4 extracellular domain to inhibit Lgr4 signaling had lower osteoporosis induced by RANKL injection or ovariectomy,^9,219^ suggesting this GPCR as a potentially valuable therapeutic target in several bone diseases.

Deletion of 16 δ-group GPCR genes caused bone dysfunctions in mouse models: deficiency of *Ebi2*,^86^ *Gpr55*,^220^ *Gpr68*,^221^ *P2y6*,^222^ and *Ptafr*^42^ increased mouse bone mass and BMD; while *Gpr65*,^223^ *Gpr103*,^224^ *Lgr4*,^8,9^ *P2y1*,^225^ *Rxfp2*,^211,215^ *Tshr*^37^ reduced bone mass and BMD; and *P2y12*^–/–^ mice had reduced age-associated bone loss with lower osteoblast activity,^226^ while deletion of *Par2*^227^ bone prevented periodontal disease in mice. Defective *Ebi2* signaling suppressed osteoclast precursor cell migration to bones, which led to increased male mouse bone mass and protection of female mice from osteoporosis due to age or estrogen deficiency.^86^ *Gpr55*^−/−^ mice had a significant increase in BMD due to stimulated osteoclast function,^220^ and BMD was increased in *Gpr68*^−/−^ mice by increasing bone turnover and a shift toward increased bone formation over resorption.^221^ The long bones and spine in *P2y6r*^−/−^ mice exhibited increased bone mineralization, cortical bone volume, and cortical thickness caused by suppressing osteoclastogenesis, whereas trabecular bone parameters were unaffected.^222^ Hikiji et al.^42^ found that *Pafr* knockout suppressed bone resorption, thus preventing bone loss in ovariectomized (OVX) mice. In contrast, *Gpr65*^−/−^ mice had elevated OVX-induced bone loss induced with enhanced osteoclast formation and osteoclastic calcium resorption.^223^ *Gpr103*^−/−^ mice had lower trabecular bone density, possibly from suppressing osteoblast-mediated bone formation, and the kyphosis phenotype was also found in *Gpr103* knockout female mice.^224^ *P2y1* deletion reduced mouse BMD in part through increasing osteoclast formation and activity via ATP and ADP.^225,228^ *Rxfp*-deficient mice presented with lower bone mass and a reduction in bone turnover via disrupted regulation of osteoblastogenesis and osteoclastogenesis.^211,215^ The BMD reduction in *Tshr*^−/−^ mice was caused by altering the regulation of both bone formation and resorption.^37^ Keratinocyte-specific deletion of *Par2* prevented periodontal bone loss by suppressing the inflammatory cascade, ultimately inhibiting osteoclast differentiation and activity.^227^ *Tshr* knockout mice only reduced femur length,^37^ while *P2y13*^−/−^ mice had increased tibia and tail length,^229^ and *Par2* deletion alleviated mouse arthritis.^230^

Furthermore, several GPCR gene knockout mice displayed different phenotypes in different strains. The bone mass was reduced in young (4-week-old) *P2y13*-knockout mice via promotion of osteoblastogenesis and suppression of osteoclastogenesis, but mature (>10-week-old) *P2y13*-knockout mice showed the opposite bone phenotype via suppression of osteoblastogenesis.^229,231^ *P2y2* deficiency increased mouse bone mass in C57BL/6 mice^225,232^ by promoting bone reformation and suppressing bone resorption but exhibited reduced bone mass in SV129 mice^233^ by reducing osteoblast differentiation and mineralization. *P2y7* knockout reduced bone mass in mixed genetic mice (129/OlaXC57BL/6XDBA/2) by reducing osteoblast number and activity^234^ but increased cortical thickness in C57 mice^235^ promoting osteoclast-mediated bone resorption (Table 5).

### Adhesion family

The adhesion GPCR family, including 33 human and 31 mouse GPCRs^236^ (also referred to as family B^45^, B2,^237^ EGF-TM7 receptors,^238^ or the LNB-TM7 family^239^), is the second largest subgroup of GPCRs. The adhesion GPCRs are divided into nine distinct subfamilies that share typical adhesion GPCR features.^240^ The nine subfamilies are *ADGRL* (latrophilins), *ADGRA*, *ADGRC* (*CELSR*s), *ADGRD*, *ADGRG*, *ADGRV* (*GPR98*), *ADGRE* (*EGF-TM7*), *ADGRF*, and *ADGRB* (*BAI*s).^236^ Adhesion GPCRs typically have an extensive N-terminal extracellular region featuring various domains that interact with the extracellular environment to execute adhesive functions.^241^ Each receptor subfamily has a specific combination of domains in its N-terminal extracellular region. Receptors within a subfamily have differing numbers of domain repeats, with consequent variation in their N-terminal extracellular region.^241^

A feature unique to adhesion family GPCRs is their autoproteolytic cleavage at the GPCR proteolysis site,^242,243^ which occurs in the conserved GPCR autoproteolysis-inducing (GAIN) domain.^244,245^ Autoproteolysis splits the highly glycosylated N-terminal fragment (NTF) from the membrane-spanning C-terminal fragment (CTF), which contains the canonical 7TM domain and the intracellular domain. The extracellular NTFs function similar to adhesion proteins, while CTFs activate intracellular signaling cascades.^240^ Adhesion GPCRs are essential components in developmental processes.^246^ Human adhesion GPCR mutations take part in nervous, bone, and cardiovascular disorders and cancers of all major tissues.^247–249^

Analysis of human adhesion GPCR SNPs revealed four GPCRs that were associated with human bone diseases or dysfunctions. However, only two adhesion GPCR knockout animal models with bone phenotypes have been reported. The mutation of *GPR126* was associated with alterations in AIS,^248,250–253^ human height,^253–257^ arthrogryposis multiplex congenital,^258^ and aggressive periodontitis.^259^ Xu et al.^252^ found that three intronic SNPs of *GPR126* (rs6570507, rs7774095, and rs7755109) were significantly associated with AIS in Chinese populations, and Kou et al.^253^ also found that rs6570507 was the most significantly linked SNP to AIS in Japanese and European ancestry populations. Liu et al. found that SNPs rs6570507, rs3748069, and rs4896582 were associated with human height in Australian twin families,^256^ and rs6570507 was also correlated with trunk length in a European GWAS meta-analysis.^257^ Ravenscroft et al.^258^ found that a missense substitution (p. Val769Glu [c.2306T>A]) impaired GPR126 autoproteolytic cleavage, resulting in reduced peripheral nerve myelination, possibly causing severe arthrogryposis multiplex congenital, and Kitagaki et al.’s study^259^ in the Japanese population found that the *GPR126* SNP rs536714306 impairs signaling and BMP2, ID2, and ID4 expression, negatively influences periodontal tissue, and leads to aggressive periodontitis, suggesting that bearers have an elevated risk for aggressive periodontitis. High *GPR56* expression is correlated with positive rheumatoid factor levels in rheumatoid arthritis patients^260^ and with the proliferation and invasion capacity of osteosarcoma cells.^261^ Liu et al. found that knockdown of *GPR110* can decrease human osteosarcoma cell proliferation, migration, and invasion capacity, suggesting a role of *GPR110* in tumor progression and possible value as a novel prognostic biomarker in osteosarcoma.^262^ Finally, Tonjes et al. found that two *GPR133* variants (rs1569019 and rs1976930) were linked to adult height in Sorbian individuals,^263^ in accord with a study that reported a microdeletion at 12q24.33, approximately 171.6 kb downstream of *GPR133*, which influences height in the Korean population.^264^

In animal models, cartilage tissue-specific *Gpr126* deletion caused idiopathic scoliosis and pectus excavatum accompanied by annulus fibrosis development in the intervertebral discs and increased chondrocyte apoptosis. Gpr126 was postulated to signal via upregulation of *Gal3st4* transcription without altering intracellular cAMP.^253,265^ Furthermore, *Cd97* deficiency increased mouse bone mass, decreased osteoclast number,^266^ and reduced arthritis^267^ (Table 6).

**Table 6:** Bone diseases or dysfunctions caused by adhesion GPCR mutation or deletion

GPCR	Species	Bone diseases or dysfunctions caused by GPCR mutation or deletion	References
CD97	Mouse	Increased bone mass reduced arthritis	Yeon et al.^266^ Hoek et al.^267^
GPR56	Human	High levels were associated with rheumatoid factor and osteosarcoma proliferation and invasion	Tseng et al.^260^ Chen et al.^261^
GPR110	Human	Prognostic biomarker in osteosarcoma	Liu et al.^262^
GPR126	Human	rs6570507, rs7774095, and rs7755109 SNPs were associated with AIS	Qin et al.^250^ Ikegawa et al.^248^ Giampietro^251^ Xu et al.^252^ Kou et al.^253^ Soranzo et al.^257^
		rs6570507, rs3748069, and rs4896582 SNPs were associated with reduced height	Karnik et al.^254^ Liu et al.^256^ Soranzo et al.^257^
		The missense substitution (p.Val769Glu [c.2306T>A]) may be caused by severe arthrogryposis multiplex congenita	Ravenscroft et al.^258^
		The rs536714306 SNP was associated with aggressive periodontitis	Kitagaki et al.^259^
	Mouse	Induced idiopathic scoliosis and pectus excavatum	Karner et al.^265^
GPR133	Human	The rs1569019 and rs1976930 SNPs were associated with adult height	Kim et al.^264^ Kim et al.^249^ Tonjes et al.^263^

*AIS* adolescent idiopathic scoliosis, *BMD* bone mineral density, *GPCR* G protein-coupled receptor, *SNP* single-nucleotide polymorphism

### Frizzled/Taste2 family

The Frizzled/Taste2 receptors span two distinct clusters: the frizzled receptors (11 in both humans and mice) and the TAS2 receptors (25 human and 34 mouse).^46,268^ Although obvious receptor similarities between these different branches are lacking, several features that differ from the other four GPCR families are shared among the sequences from this family of GPCRs, for example, IFL in TM2, SFLL in TM5, and SxKTL in TM7. The Frizzled receptors are highly conserved evolutionarily, while Taste2 GPCRs probably rapidly evolved and expanded in number.^47^ The ten Frizzled receptors, FZD1–10, plus SMOH, are conserved in most mammals, with highly similar primary amino acid sequences, making the Frizzled family the most highly conserved GPCR family.^269,270^ Frizzled GPCRs are Wnt receptors that play key roles in organism development, diseases and cell signaling.^271–277^ Frizzled GPCRs have a CRD/FZ or FZ domain with ten conserved cysteines. The TAS2 receptors are not related to the glutamate receptor family’s TAS1 receptors. TAS2 receptors have seven hydrophobic regions considered putative TM domains, but their very short N-terminal regions are unlikely to bind ligands.^278^ All 25 functional human TAS2 genes (hT2Rs) are expressed in taste receptor cells of the human gustatory papilla.^279^ DNA polymorphisms in 25 functional hT2R genes are relatively common, featuring a large number of amino acid substitutions.^280,281^

Analysis of the human Frizzled/Taste2 family GPCR SNP revealed three GPCRs that were associated with human bone diseases or dysfunctions, and only three GPCR knockout animal models with bone phenotypes have been reported to date. Two *FZD1* promoter SNPs (rs2232157, rs2232158) were linked to femoral neck area BMD in men of African ancestry.^282,283^ *FZD6* sequencing revealed homozygosity for a nonsense mutation (c.1750G>T [p. Glu584X] and a missense mutation (c.1531C>T [p. Arg511Cys]) causes isolated autosomal-recessive nail dysplasia.^284–286^ Mutation of *frizzled-9* was associated with reduced human BMD.^273,287^

Furthermore, Frojmark et al. reported that approximately 50% of male *Fzd6*^−/−^ mice displayed abnormal claw morphology or lack of claws, potentially by suppressing either WNT-3A-FZD or WNT-5A-FZD signaling.^284^ Curiously, this phenotype was absent in female mice. *Frizzled-9* knockout induced mouse osteopenia by reducing osteoblast-mediated bone formation^288^ and reduced new bone formation after fractures by disturbing osteoblast function.^289^ *Smoh* knockout reduced BMD, body length, and bone callus formation by reducing osteogenic differentiation in mice^38,290^ (Table 7).

**Table 7:** Diseases or dysfunctions caused by Frizzled/Taste2 GPCR mutation or deletion

GPCR	Species	Bone diseases or dysfunctions caused by GPCR mutation or deletion	References
Frizzled-1	Human	rs2232157 and rs2232158 SNPs were associated with reduced BMD	Zhang et al.^282^ Yerges et al.^283^
Frizzled-6	Human	Two mutations (c.1750G>T and c.1531C>T) caused nail dysplasia	Frojmark et al.^284^ Wilson et al.^285^ Naz et al.^286^
	Mouse	50% of male mice displayed abnormal claw morphology or lack of claws	Frojmark et al.^284^
Frizzled-9	Human	The mutation was associated with reduced BMD	Francke et al.^287^ Wang et al.^273^ Heilmann et al.^289^
	Mouse	Induced osteopenia and reduced formation of new bone after fractures	Albers et al.^288^
SMOH	Mouse	Reduced BMD, body length, and bone callus formation	Cho et al.^38^ Wang et al.^290^

*BMD* bone mineral density, *GPCR* G protein-coupled receptor, *SNP* single-nucleotide polymorphism

### Secretin family

The secretin receptor family has 15 members divided among four subgroups: CRHRs/CALCRLs, PTHRs, GLPRs/GCGR/GIPR, and GHRHR/PACAP/SCTR/VIPR.^46^ These GPCRs are characterized by six conserved N-terminal domain cysteines and by seven conserved TM helices.^291–293^ The N-terminal extracellular domain recognizes the secretin C-terminus,^291,294,295^ with the conserved cysteines required for receptor function.^296^ The secretin family GPCRs bind paracrine or endocrine peptide hormones (typically 30–40 amino acids long^297^), often indiscriminately. Secretin GPCRs regulate diverse physiological responses, including the cell cycle, differentiation, proliferation, and additional endocrine hormone release. Secretin GPCRs generally signal through AC and to a lesser extent through PLC and intracellular calcium mobilization, although they are not confined to these pathways.^298^ Currently used drugs against osteoporosis, hypercalcemia, Paget’s disease, type II diabetes, depression, anxiety, and pancreatic diseases operate by modulating secretin GPCRs.

Five mutations or deletions in secretin family GPCRs were associated with human bone diseases or animal bone dysfunctions. A *CALCR* SNP was associated with BMD, bone mass, and fracture risk.^299–303^ Multiple reports connected a Pro447Leu (rs1801197) polymorphism of *CALCR* and osteoporosis-related phenotypes and fracture risk in postmenopausal women,^299,301–306^ and an intronic SNP of rs2051748 was also significantly associated with vertebral trabecular BMD in older Caucasian men.^300^ Zupan et al. found that there was a higher expression of *CALCR* in osteoarthritic patients.^299^ Furthermore, *Calcr*^+/−^ mice have a high bone mass with increased bone formation.^307^ Rivadeneira et al. found that the rs9303521 SNP *CRHR1* was associated with lumbar spine BMD in people of Northern European descent.^308^ Several studies inferred that the *GHRHR* SNPs rs17159772, rs4988494, rs2267721, rs4988498, and rs4988505 were associated with reduced human height, indicating that *GHRHR* might affect normal human height variation.^309–312^ Furthermore, the phenotype of pituitary dwarfism was also observed in individuals with *GHRHR* mutations (IVS1 + 1G→A or IVS8+1G>A).^313–318^ Harsloef et al. and Torekov and colleagues reported that the *GIPR* polymorphism Glu354Gln (rs1800437) was associated with reduced human BMD and bone mass and increased fracture risk.^319,320^

PTHR is the most extensively studied GPCR in bone development and disease. The *PTHR* SNPs rs1531137, rs1869872, rs4683301, and rs724449 were associated with reduced human height,^321–323^ BMD,^321–324^ and chondrodysplasia.^325,326^ Consistently, *Pthr* knockout mice had reduced body length and limbs,^327–329^ reduced trabecular BMD and osteocyte number, delayed ossification, and reduced chondrocyte proliferation and differentiation,^39,329–333^ with increased cortical bone thickness.^39,334,335^ PTH is a systemic hormone that regulates calcium homeostasis and bone remodeling by activating PTHR.^329,335^ It can activate Gs and Gq, leading to cAMP production, PKA activation and stimulation of phospholipase for PKC activation to stimulate downstream signaling events.^336^ The 1–34 amino acid peptide of PTH (PTH(1–34)) is an anti-osteoporosis drug that functions by stimulating osteoblast proliferation,^337^ increasing osteoblast activity,^338^ and protecting osteoblasts from apoptosis^339^ through direct binding to PTHR.^340^ Interestingly, PTH(1–34) also maintains intervertebral disc homeostasis during aging, suggesting that *PTH* has the ability to maintain skeletal homeostasis^341^ (Table 8).

**Table 8:** Bone diseases or dysfunctions caused by secretin GPCR mutation or deletion

GPCR	Species	Bone diseases or dysfunctions caused by GPCR mutation or deletion	References
CALCR	Human	SNPs rs1801197 and rs2051748 were associated with BMD and fracture risk; there is a higher expression of *CALCR* in osteoarthritis	Zupan et al.^299^
			Zmuda et al.^300^
			Lee et al.^301^
			Masi et al.^302^
			Zofkova et al.^303^
	Mouse	Increased bone mass	Dacquin et al.^307^
CRHR1	Human	rs9303521 SNP was associated with BMD	Rivadeneira et al.^308^
GHRHR	Human	SNPs rs17159772, rs4988494, rs2267721, rs4988498, and rs4988505 were associated with reduced height	Aguiar et al.^309^
			Camats et al.^310^
			Inoue et ai.^311^
			Martari et al.^312^
		Mutations of IVS1 + 1G→A or IVS8+1G>A were associated with dwarfism	Wang et al.^313^
			Oliveira et al.^314^
			Salvatori et al.^315^
			Baumann^316^
			Baumann et al.^317^
			Wajnrajch et al.^318^
GIPR	Human	SNP rs1800437 was associated with lower BMD and bone mass and increased fracture risk	Harsloef et al.^319^
			Torekov et al.^320^
	Mouse	Reduced BMD, bone mass, and bone strength and promoted bone resorption	Xie et al.^342^
			Yamada et al.^343^
			Mieczkowska et al.^40^
			Tsukiyama et al.^344^
			Shen et al.^345^
PTHR	Human	SNPs rs1531137, rs1869872, rs4683301, and rs724449 were associated with reduced height, BMD, and chondrodysplasia	Scillitani et al.^321^
			Zhang et al.^322^
			Vilarino et al.^323^
			Wynne et al.^324^
			Schipani et al.^325^
			Karaplis et al.^326^
	Mouse	Reduced body and mouse limb length	Qiu et al.^329^
			Lanske et al.^327^
			Hirai et al.^328^
		Delayed ossification and reduced chondrocyte proliferation and differentiation	Qiu et al.^329^
			Guo et al.^330^
			Lanske et al.^39^
			Lanske et al.^331^
			Karperien et al.^332^
			Hopyan et al.^333^
		Lower trabecular BMD and osteocyte number and increased cortical bone thickness	Qiu et al.^335^
			Lanske et al.^39^
			Powell et al.^334^

*BMD* bone mineral density, *GPCR* G protein-coupled receptor, *SNP* single-nucleotide polymorphism

### Other 7TM receptors

Several 7TM receptors did not fit into any family/group/cluster of the GRAFS classification system; therefore, these receptors are called other 7TM receptors. Most of them are orphan GPCRs.^46,47,268,275^ There are five genes associated with bone diseases or dysfunctions in humans or mice from the other 7TM receptor group.

*GPR22* is an orphan GPCR. In silico and in vitro experiments suggested that the T-alleles of the rs3757713 and rs3815148 SNPs were associated with *GPR22* expression in lymphoblasts. GPR22 was detected in cartilage and osteophytes in OA-induced mouse models but not in normal cartilage. Kerkhof et al.^346^ identified SNP rs3815148 (located close to the *GPR22* gene) as an OA susceptibility locus in a large association analysis of OA genetics with 14 938 OA cases and approximately 39 000 controls. Verleyen et al. found that altering the expression of *Gpr22* in zebrafish embryos induced a downward-curving tail, which is often associated with defects in ciliogenesis.^347^

GPR177, which is similar to the Frizzled family of GPCRs, is a Wnt signaling pathway component^348^ involved in bone cell differentiation. As part of the RANK pathway, the gene positively regulates the NF-κB cascade.^349^ Several multistage genome-wide association study meta-analyses identified four loci (rs1430742, rs2566755, rs2772300, and rs6588313 SNPs) in *GPR177* that were associated with human lumbar spine, femoral neck, or total hip BMD.^308,350–353^ Zhong et al. found that deletion of *Gpr177* in mice resulted in bone loss, increased bone resorption, and defects in chondrogenesis and ossification^354,355^ (Table 9).

**Table 9:** Bone diseases or dysfunctions caused by other 7TM receptor mutations or deletions

GPCR	Species	Bone diseases or dysfunctions caused by GPCR mutation or deletion	References
GPR22	Human	Associated with osteoarthritis	Kerkhof et al.^346^
	Zebrafish	Induced curvature of the tail	Verleyen et al.^347^
GPR30	Mouse	Increased male bone mass and reduced female femur length	Ford et al.^41^ Martensson et al.^364^
GPR39	Mouse	Increased bone formation and osteoblast differentiation	Jovanovic et al.^356^
GPR40	Mouse	Reduced BMD, bone mass, and aggravated osteoarthritis-induced phenotype	Wauquier et al.^43^ Monfoulet et al.^375^
GPR177	Human	Associated with reduced BMD	Rivadeneira et al.^308^ Deng et al.^350^ Roshandel et al.^351^ Styrkarsdottir et al.^352^ Hsu et al.^353^
	Mouse	Reduced bone mass and increased bone resorption	Zhong et al.^354^
		Defects in chondrogenesis and ossification	Zhong et al.^355^

*BMD* bone mineral density, *GPCR* G protein-coupled receptor

The deletion of either *Gpr30*^41^ or *Gpr39*^356^ increased bone mass in mice, but in contrast, the deletion of *Gpr40*^43^ or *Gpr177*^354^ reduced mouse bone mass and BMD. GPR30, as an estrogen receptor, is activated by estrogen and the GPR30-specific agonist G1.^357^ GPR30 activation elevates cAMP levels, intracellular Ca^+^^2^ mobilization, and transactivation of epidermal growth factor receptors.^358–361^ *GPR30* expression in human bone is limited to osteoblasts, osteocytes, and osteoclasts.^362^ In immortalized rat skull preosteoblasts, *Runx2* upregulated *Gpr30* gene expression and increased osteoblast progenitor proliferation, suggesting that Gpr30 may promote osteoblast differentiation.^363^ Confounding this, however, Ford et al. reported that *Gpr30* loss increased bone mass, mineralization, and growth plate proliferation in male mice,^41^ whereas Martensson et al.^364^ reported that *Gpr30* deletion reduced female mouse femur length.

Gpr39 is a zinc-sensing receptor that is expressed by osteoblast cell lines.^365^ Zinc potently and specifically activates Gpr39 to induce Gq, G12/13, and Gs pathway signaling, suggesting that zinc is a physiologically important agonist.^366^ Jovanovic et al.^356^ found that *Gpr39*-deficient mice have higher bone stiffness and a higher mineral-to-matrix ratio, along with increased bone formation and osteoblast differentiation, suggesting that zinc sensing by *Gpr39* is important in regulating collagen processing and mineralization, which are required for the proper maintenance of bone integrity.

GPR40 is highly expressed in pancreatic beta cells, where it interacts with medium-to-long chain fatty acids,^367–369^ to potentiate glucose-induced insulin secretion.^370^ *GPR40* is also expressed in leukocytes, osteoclasts, and monocytes.^371,372^ Cornish et al.^373^ observed that a GPR40 agonist inhibits osteoclastogenesis, which is similar to the effects of free fatty acids. Furthermore, *Gpr40* downregulation protects osteocytes from apoptosis.^374^ Wauquier et al.^43^ observed that *Gpr40*^−/−^ mice had a reduction in BMD and bone mass with higher promoting osteoclast differentiation, and Monfoulet et al.^375^ observed a more severe OA-induced phenotype in *Gpr40*^−/−^ mice, marked by elevated tidemark exposure, osteophyte formation, and subchondral bone sclerosis (Table 9).

## Conclusions

GPCRs play crucial roles in bone development, remodeling, and diseases by activating GPCR signaling pathways. Our results show that 92 receptors (5 glutamate family, 67 rhodopsin family, 5 adhesion, 4 frizzled/taste2 family, 5 secretin family, and 6 other 7TM reporters) were associated with bone diseases and dysfunctions (35 in humans and 72 in animals), and the catalog of diseases linked to GPCR malfunction continues to expand.

In summary, the GPCR superfamily plays a key role in regulating bone diseases and remodeling. Different GPCRs from different subfamilies may have similar physiological functions to regulate these processes; however, the same GPCR may have different physiological functions in different populations or animal models. Although the field has made significant progress in understanding how GPCRs influence bone development and diseases, much remains unknown. Since many GPCR mutations are embryonic lethal, the availability of mouse models to study GPCRs has been a significant barrier to progress. Fortunately, conditional knockout approaches have proven effective in many cases, allowing characterization of the detailed mechanisms involving GPCRs in bone diseases and dysfunctions. This should allow enormous advances in translational medicine, as GPCRs are generally regarded as a superb class of drug targets.
